# The International Journal of Public Health

**Published:** 1921-06-25

**Authors:** 


### The International Journal of Public Health.

We have received a letter from the League of Red Cross Societies in which they ask us to make it clear that the article on the treatment of venereal disease, by Dr. Arthur Vernes, which we quoted in these columns on June 4, appeared in the *International Journal of Public Health.* We referred then to Dr. Vernes' original treatise on the subject, but the source of his article in the *International Journal* was undoubtedly omitted. We gladly make good this omission. The organ of the League is doing so great a service to the cause of public health throughout, the world that it deserves all the recognition it is possible to give it. We have repeatedly brought before the notice of our readers the value to all public health authorities and to the medical profession of this carefully compiled and comprehensive publication, and welcome the opportunity of doing so again.

